# Robust Trajectory Tracking Control for Variable Stiffness Actuators With Model Perturbations

**DOI:** 10.3389/fnbot.2019.00035

**Published:** 2019-06-11

**Authors:** Zhao Guo, Jiantao Sun, Jie Ling, Yongping Pan, Xiaohui Xiao

**Affiliations:** ^1^School of Power and Mechanical Engineering, Wuhan University, Wuhan, China; ^2^Wuhan University Shenzhen Research Institute, Shenzhen, China; ^3^School of Data and Computer Science, Sun Yat-sen University, Guangzhou, China; ^4^National University of Singapore (Suzhou) Research Institute, Suzhou, China

**Keywords:** variable stiffness actuator, nonlinear disturbance observer, compliant actuator, feedback linearization, composite control, model perturbations

## Abstract

Variable Stiffness Actuators (VSAs) have been introduced to develop new-generation compliant robots. However, the control of VSAs is still challenging because of model perturbations such as parametric uncertainties and external disturbances. This paper proposed a non-linear disturbance observer (NDOB)-based composite control approach to control both stiffness and position of VSAs under model perturbations. Compared with existing non-linear control approaches for VSAs, the distinctive features of the proposed approach include: (1) A novel modeling method is applied to analysis the VSA dynamics under complex perturbations produced by parameter uncertainties, external disturbances, and flexible deflection; (2) A novel composite controller integrated feedback linearization with NDOB is developed to increase tracking accuracy and robustness against uncertainties. Both simulations and experiments have verified the effectiveness of the proposed method on VSAs.

## Introduction

Recently, compliant robots have attracted increasing attention in the robotics community. Variable stiffness actuators (VSAs), a kind of compliant actuators, have been introduced to develop new-generation robots because of its abilities to increase safety in human-robot interaction, to satisfy dynamic requirements, and to provide adaptability in unknown environments (Vanderborght et al., 2013; Grioli et al., 2015; Guo et al., 2015; Wolf et al., 2016; Pan et al., 2017). VSAs are usually multi-input multi-output (MIMO) non-linear systems, where the stiffness and position of the VSAs can be adjusted simultaneously by decoupling control methods (Kim and Song, 2012). However, in these actuators, the stiffness variation brings physical modifications, which requires the controllers to transit among different working conditions quickly. The physical coupling between stiffness and position mechanisms also introduces undesired complexity to control systems (Jafari, 2014). Furthermore, the performances of these actuators are severely affected by parametric uncertainties and external load perturbations, especially during interacting with environments. Therefore, it is essential to develop advanced control strategies for VSAs used in robotic systems.

Different control approaches have been proposed to for VSAs. The PD-based control is a simple and easy method to regulate position and stiffness of VSAs simultaneously. However, PD parameters should be tuned manually to obtain good tracking accuracy in different stiffness condition. Recently, a feedback linearization technique was also exploited for the control of VSAs in Palli et al. (2008) and Buondonno and De Luca (2016). This technique requires significant efforts in system modeling as well as the identification of the system parameters. In addition, a control strategy with fixed gains can cause limited performance in the dynamic variations of the VSAs (Buondonno and De Luca, 2016). To improve the control performance, other advanced control approaches, such as backstepping control (Petit et al., 2015), gain-scheduling control (Sardellitti et al., 2013), non-linear model predictive control (Zhakatayev et al., 2015), adaptive neural network control (Guo et al., 2017), and prescribed performance control (Psomopoulou et al., 2015), have been proposed for VSAs. Although these control approaches have been proved to be effective to improve tracking performances of VSAs, they have a significant limitation that the performances heavily depend on exact models of VSAs (Palli and Melchiorri, 2011; Petit and Albu-Schaffer, 2011). In addition, the disturbance rejection ability of these controllers is achieved by sacrificing the nominal control performance. A novel approach has been proposed to the control of VSA actuated robots, aiming to preserve their dynamic behavior which has been obtained because of the elastic element in the robot structure (Della Santina et al., 2017; Keppler et al., 2018). Furthermore, a decentralized, iteratively learned feedforward approach, combined with a locally optimal feedback control has been introduced in (Angelini et al., 2018). The effectiveness of the method is experimentally verified on several robotic structures and working conditions.

Disturbance observer (DOB)-based control is promising to reject disturbances and to improve robustness against modeling uncertainties (Roozing et al., 2016). This approach has been adopted in the control of serial elastic actuators (SEAs). For instance, a linear DOB-based control method was used for the prismatic SEA to achieve high precision force control in Park et al. (2017). However, this method cannot be directly applied to control VSAs because of non-linearities and model uncertainties. This paper introduces a non-linear disturbance observer (NDOB)-based composite controller to improve the control performance and reject load disturbances for a new type of serial VSA (SVSA), in which stiffness and position can be separately controlled by two motors with a series configuration (Sun et al., 2017, 2018a,b). In the proposed control framework, a NDOB is applied to estimate disturbances so as to enhance the disturbance rejection ability. Based on feedback linearization, a composite control law is developed to stabilize the non-linear dynamics. It is proven that the proposed controller can eliminate external disturbances by a proper selection of the compensation gain. The major contributions of this study include: (i) Different from exising VSA models, the SVSA model in this study considers the composite disturbances produced by system uncertainties, flexible effects, and external disturbances; (ii) A novel disturbance compensation method is developed to attenuate model perturbations for the control of SVSAs; (iii) Experimental studies have been carried out to demonstarete effectivencess and robustness of the proposed controller for SVSAs. In our previous work (Guo et al., 2018), we introduced a NDOB-based control (NDOBC) method for SVSAs, and conducted basic experiments related to position and theoretical stiffness tracking. The current work extends our previous work in terms of dynamic modeling and real-time control of SVSAs. We conduct both simulations and experiments comparing our approach with a feedback linearization-based controller.

The remainder of this paper is organized as follows. Section Actuator Dynamics and Problem Formulation introduces the SVSA dynamics and formulates the control problem. Section Non-Linear Disturbance Observer-Based Control describes the proposed NDOBC design and the control system stability issue. Section Simulation Results shows simulation and experimental results of the proposed controller. Section Experimental Results draws the conclusion of this study.

## Actuator Dynamics and Problem Formulation

In this section, the SVSA model is presented firstly. Subsequently, by considering parametric variations and external disturbances acted on the actuator, the control problem is formulated.

### Actuator Dynamics

**Figure 1:**
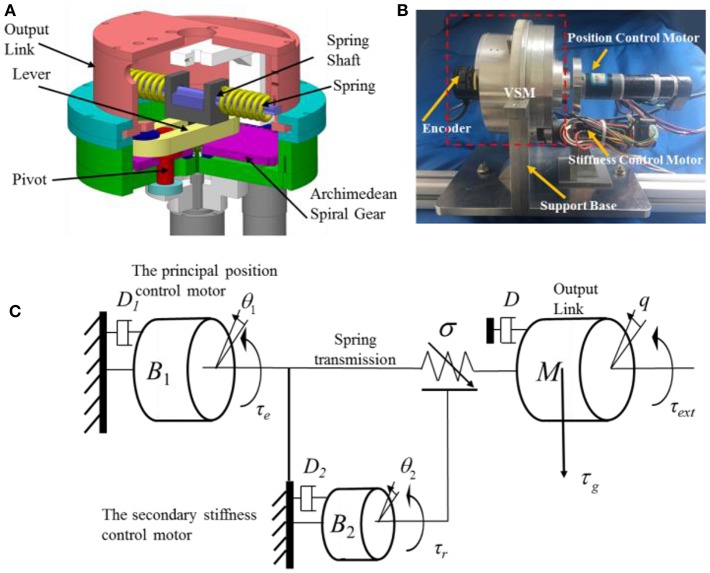
The CAD model **(A)**, prototype **(B)**, and schematic model **(C)** of the SVSA.

A novel SVSA based on an Archimedean spiral relocation mechanism (ASRM) was developed in Sun et al. (2017). As illustrated in Figure 1, this SVSA consists of a variable stiffness mechanism (VSM), a principal motor and a secondary motor, where the principal motor drives the output link motion through the spring transmission, and the secondary motor adjusts the actuator theoretical stiffness by the ASRM. Figure 1 shows the CAD model, prototype, and schematic model of the SVSA.

By considering gravity and external loads, the SVSA dynamics can be represented as follows:

(1) $$\{\begin{array}{l} M\ddot{q}+D\dot{q}+\tau_{e}(\theta_{2},\varphi )+\tau_{g}(q)=\tau_{ext}\\ B_{1}{\ddot{\theta }}_{1}+D_{1}{\dot{\theta }}_{1}-\tau_{e}(\theta_{2},\varphi )=u_{1}\\ B_{2}{\ddot{\theta }}_{2}+D_{2}{\dot{\theta }}_{2}+\tau_{r}(\theta_{2},\varphi )=u_{2} \end{array}$$

where *q* is a position of the output link, θ_*i*_ with *i* = 1, 2 is the angle position of each motor, φ: = *q* − θ_1_ is a deflection angle of the elastic transmission, *M* is an inertia of the output link, *B*_*i*_ is a reflected inertia of each motor, *D* is a reflected damping of the link, *D*_*i*_ is a reflected damping of each motor, τ_*g*_(*q*) is a gravity torque, τ_*r*_ is a coupling reaction torque, τ_*e*_ is an elastic torque of the spring transmission, *u*_*i*_ is a control input of each motor, and τ_*ext*_ is an external torque. The general specifications are shown in Table 1.

**Table 1:** Parameter specifications of the SVSA.

**Description**	**Symbol (unit)**	**Value**
Output link inertia	M (kgm^2^)	0.0103
Motor M1 + gearbox + intermediate connecter Inertia	B_1_ (kgm^2^)	0.0234
Motor M2 + gearbox + ASAM Inertia	B_2_ (kgm^2^)	0.014
Output link damping	D (Nms/rad)	0.005
Motor M1 damping	D_1_ (Nms/rad)	0.005
Motor M2 damping	D_2_ (Nms/rad)	0.001
Inherent spring stiffness	Ks (N/m)	1882
Radius of the actuator	R(m)	0.075
Range of motion	(deg.)	0–360
Range of deflection angle	(rad.)	0.75
Range of stiffness	(Nm/rad)	1.7–150.56

The elastic torque across the transmission is given by

(2) $$\begin{array}{l} \tau_{e}=K_{s}R^{2}\mu^{2}\frac{\sin\varphi \cos\varphi }{{\left(1-\mu \cos\varphi \right)}^{2}} \end{array}$$

where *K*_s_ is a spring stiffness, *R* is a radius of the output link, and μ is a lever length ratio. The stiffness of this SVSA is the first order of elastic torque

(3) $$\begin{array}{l} \sigma \left(\theta_{\text{2}},\varphi \right)\text{=}K_{s}R^{2}\mu^{2}\frac{\cos2\varphi -\mu \cos\varphi }{{\left(1-\mu \cos\varphi \right)}^{3}} \end{array}$$

The level length ratio μ can be written by the position of the secondary motor as follows:

(4) $$\begin{array}{l} \mu \text{=}\theta_{2}/2\pi \gamma \text{+}\mu_{0} \end{array}$$

where μ_0_ is an initial level length ratio. The coupled resistance torque, demonstrating the transmission deformation reacts on the stiffness motor, is given by

(5) $$\begin{array}{l} \tau_{\text{r}}=K_{s}R^{2}a^{2}\frac{sin2\beta sin^{2}\varphi }{2\left(R-acos\varphi \right)\left(a^{2}+R^{2}-2aRcos\varphi \right)}\; \end{array}$$

where β = arctan(−θ_2_/γ) is a tangent angle of the Archimedean Spiral gear, γ is a reduction gear ratio of the secondary motor, and *a* = μ*R* = *Rθ*_2_/2π is a distance from the pivot point to the joint center.

### Problem Formulation

Considering the parametric variations and modeling uncertainties in (1), we define the differences between the nominal and real variables as Δ*M* = *M* − *M*_*n*_, Δ*B*_1_ = *B*_1_ − *B*_1*n*_, Δ*B*_2_ = *B*_2_ − *B*_2*n*_, Δ*D* = *D* − *D*_*n*_, ΔD_1_ = *D*_1_ − *D*_1*n*_, Δ*D*_2_ = *D*_2_ − *D*_2*n*_, Δτ_e_ = τ_*e*_ − τ_*en*_, Δτ_*r*_ = τ_*r*_ − τ_*rn*_, where *M*_*n*_ is an equivalent inertia of the output link, *B*_*in*_ (*i* = 1, 2) is an equivalent reflected inertia of each motor, *D*_*n*_ is an equivalent damping of the link, *D*_*in*_ (*i* = 1, 2) is an equivalent damping of each motor, τ_*en*_ and τ_r*n*_ are nominal elastic torque and resistance torque.

Substituting these variations into (1), we wet a nominal model

(6) $$\{\begin{array}{l} (M_{n}+\Delta M)\ddot{q}+(D_{n}+\Delta D)\dot{q}+(\tau_{en}+\Delta \tau_{en})+g(q)=\tau_{ext}\\ (B_{1n}+\Delta B_{1}){\ddot{\theta }}_{1}+(D_{1n}+\Delta D_{1}){\dot{\theta }}_{1}-(\tau_{en}+\Delta \tau_{e})=\tau_{m1}\\ (B_{2n}+\Delta B_{2}){\ddot{\theta }}_{2}+(D_{2n}+\Delta D_{2}){\dot{\theta }}_{2}+(\tau_{rn}+\Delta \tau_{r})=\tau_{m2} \end{array}$$

The model uncertainties, gravity, and external disturbances are regarded as equivalent disturbances of the system:

(7) $$\{\begin{array}{l} \tau_{dis1}=\Delta M\ddot{q}+\Delta D\dot{q}+\Delta \tau_{en}+g(q)-\tau_{ext}\\ \tau_{dis2}=\Delta B_{1}{\ddot{\theta }}_{1}+\Delta D_{1}{\dot{\theta }}_{1}-\Delta \tau_{e}\\ \tau_{dis3}=\Delta B_{2}{\ddot{\theta }}_{2}+D_{2}{\dot{\theta }}_{2}+\Delta \tau_{r} \end{array}$$

Substituting Equation (7) into (6), the dynamic equations can be obtained as follows:

(8) $$\{\begin{array}{l} \;\ddot{q}=M_{n}^{-1}(-D_{n}\dot{q}-\tau_{en}-\tau_{dis1})\\ {\ddot{\theta }}_{1}=B_{1n}^{-1}(\tau_{m1}-D_{1n}{\dot{\theta }}_{1}+\tau_{en}-\tau_{dis2})\\ {\ddot{\theta }}_{2}=B_{2n}^{-1}(\tau_{m2}-D_{2n}{\dot{\theta }}_{2}-\tau_{rn}-\tau_{dis3}) \end{array}$$

The above dynamics can be rewritten in standard form

(9) $$\{\begin{array}{l} \dot{x}=f(x)+g(x)u+p(x)w\\ y=h(x) \end{array}$$

where $x={\left[q,\dot{q},\theta_{1},{\dot{\theta }}_{1},\theta_{2},{\dot{\theta }}_{2}\right]}^{T}\in R^{\text{6}}$ a states vector, $u={\left[u_{1},u_{2}\right]}^{T}$ is the control input for each motor, *y* = [*q*, σ]^*T*^ is the output position and stiffness of the actuator, and

$$\begin{array}{l} \;f\left(x\right)=\left[\begin{array}{c} \dot{q}\\ M_{{}^{n}}^{-1}\left(-D\dot{q}-\tau_{en}\right)\\ {\dot{\theta }}_{1}\\ B_{1n}^{-1}\left(-D_{1n}{\dot{\theta }}_{1}+\tau_{en}\right)\\ {\dot{\theta }}_{2}\\ B_{2n}^{-1}\left(-D_{2n}{\dot{\theta }}_{2}-\tau_{rn}\right) \end{array}\right],\\ \;g\left(x\right)=\left[\begin{array}{cc} 0 & 0\\ 0 & 0\\ 0 & 0\\ B_{1n}^{-1} & 0\\ 0 & 0\\ 0 & B_{2n}^{-1} \end{array}\right],\\ \text{and }h\left(x\right)=\left[\begin{array}{c} q\\ \sigma \end{array}\right],\\ \;u=\left[\begin{array}{c} \tau_{m1}\\ \tau_{m2} \end{array}\right],\\ \;p\left(x\right)=diag\left(p_{1},p_{2},p_{3},p_{4},p_{5},p_{6}\right)\\ \;=diag\left(0,{M_{n}}^{-1},0,B_{1n}^{-1},0,B_{2n}^{-1}\right)\in R^{6} \end{array}$$

The equilibrium point of the system (9) is ***x***_0_ = 0. Let *q*_*d*_ ∈ *R* and σ_*d*_ ∈ *R* be bounded desired outputs. Let $w={\left[w_{1},\ldots ,w_{6}\right]}^{T}=\left[\text{0,}-\tau_{dis1},0,-\tau_{dis2},0,-\tau_{dis3}\right]\in R^{6}$ be an equivalent disturbance.

This paper aims to design a NDOB-based composite control law to compensate for unknown disturbances, without knowing the exact SVSA model. The control inputs of the SVSA are from two motors, while the control outputs are the position and stiffness of the actuator.

## Non-Linear Disturbance Observer-Based Control

### Non-linear Disturbance Observer Design

A NDOB as follows is applied to compensate for the unknown disturbance in the non-linear system (9) (Chen et al., 2000; Yang et al., 2012):

(10) $$\{\begin{array}{l} {\dot{z}}_{w}=-l(x)(p(x)\lambda (x)+f(x)+g(x)u)-l(x)p(x)z_{w}\\ \hat{w}=z_{w}+\lambda (x) \end{array}$$

where *z*_*w*_ is internal state of the NDOB, and $ŵ={\left[{ŵ}_{1},\ldots ,{ŵ}_{n}\right]}^{T}$ is the estimated vector of the unknown disturbance, λ(*x*)is an intermediate variable for the observer gain *l*(*x*), which is defined as

(11) $$\begin{array}{l} l\left(x\right)=\frac{\partial \lambda \left(x\right)}{\partial x}=\left[l_{1}\left(x\right),\;l_{2}\left(x\right),\;l_{3}\left(x\right),\;l_{4}\left(x\right),\;l_{5}\left(x\right),\;l_{6}\left(x\right)\right]. \end{array}$$

We define the disturbance error *e*_*w*_ = *w* − ŵ. The estimated disturbance error of (10) is given by

(12) $$\begin{array}{l} {ė}_{w}\left(t\right)=-l\left(x\right)p\left(x\right)e_{w}\left(t\right)+ẇ \end{array}$$

**Assumption 1:** The first time derivative of the disturbance ė_*w*_ is bounded, and satisfy lim_*t* → ∞_ẇ(*t*) = 0. If the observer gain satisfies the differential equation

(13) $$\begin{array}{l} {ė}_{w}\left(t\right)\text{+}l\left(x\right)p\left(x\right)e_{w}\left(t\right)\text{=0} \end{array}$$

The estimated disturbance error (12) is locally input-to-state stable (ISS).

In order to make sure the observer error converges to 0, the observer gain is defined as

(14) $$\begin{array}{l} \begin{array}{l} l\left(x\right)=\frac{\partial \lambda \left(x\right)}{\partial x}\\ \;=diag\left(k_{w1},k_{w2},k_{w3},k_{w4},k_{w5},k_{w6}\right)\;\left(k_{wi}>0,1\leq i\leq 6\right) \end{array} \end{array}$$

We define the intermediate variable λ(*x*) as

(15) $$\begin{array}{l} \lambda \left(x\right)=diag\left(k_{w1},k_{w2},k_{w3},k_{w4},k_{w5},k_{w6}\right)x \end{array}$$

Thus, the state equation of the disturbance observer is given by

(16) $$\{\begin{array}{l} {\dot{z}}_{w1}=-k_{w1}x_{2}\\ {\dot{z}}_{w2}=-M^{-1}k_{w2}z_{w2}-k_{w2}(M^{-1}k_{w1}x_{2}+M^{-1})(-Dx_{2}-\tau_{e})\\ {\dot{z}}_{w3}=-k_{w3}x_{4}\\ {\dot{z}}_{w4}=-B_{1}^{-1}k_{w4}z_{w4}-k_{w4}(B_{1}^{-1}k_{w4}x_{4}+B_{1}^{-1}(-D_{1}x_{4}+\tau_{e})\\ \;+B_{1}^{-1}u_{1})\\ {\dot{z}}_{w5}=-k_{w5}x_{6}\\ {\dot{z}}_{w6}=-B_{2}^{-1}k_{w6}z_{w6}-k_{w6}(B_{2}^{-1}k_{w6}x_{6}+B_{2}^{-1}(-Dx_{6}-\tau_{r})\\ \;+B_{2}^{-1}u_{2}) \end{array}$$

### Feedback Linearization

**Definition:** The vector relative degree of the system (9) is (*r*_1_, *r*_2_) at the equilibrium *x*_0_ if $L_{g_{j}}L_{f}^{k}h_{i}\left(x\right)=0$ (1 ≤ *j* ≤ 2, 1 ≤ *i* ≤ 2) for all *x* in a neighborhood of *x*_0_ and all *k* < *r*_*i*_ − 1, thus the matrix

(17) $$\begin{array}{l} A\left(x\right)=\left[\begin{array}{cc} L_{g_{1}}L_{f}^{r_{1}-1}h_{1} & L_{g_{2}}L_{f}^{r_{1}-1}h_{1}\\ L_{g_{1}}L_{f}^{r_{2}-1}h_{2} & L_{g_{2}}L_{f}^{r_{2}-1}h_{2} \end{array}\right] \end{array}$$

is non-singular at *x* = *x*_0_. The input relative degree of (9) is calculated as *r* = [4, 2] with *n* = *r*_1_ + *r*_2_, so (9) can be linearized. Thus, *A*(*x*) can be rearranged as

(18) $$\begin{array}{l} A\left(x\right)=\left[\begin{array}{cc} L_{g_{1}}L_{f}^{\text{3}}h_{1} & L_{g_{2}}L_{f}^{\text{3}}h\\ L_{g_{1}}L_{f}h_{2} & L_{g_{2}}L_{f}h_{2} \end{array}\right] \end{array}$$

A new coordinate transformation for feedback linearization is define as follows:

(19) $$\begin{array}{l} \Phi \left(x\right)=\xi \end{array}$$

where

(20) $$\begin{array}{l} \xi^{i}=\left[\begin{array}{c} \xi_{1}^{1}\\ \xi_{2}^{1}\\ \xi_{3}^{1}\\ \xi_{4}^{1}\\ \xi_{1}^{2}\\ \xi_{2}^{2} \end{array}\right]=\left[\begin{array}{c} h_{1}\left(x\right)\\ L_{f}h_{1}\left(x\right)\\ L_{f}^{2}h_{1}\left(x\right)\\ L_{f}^{3}h_{1}\left(x\right)\\ h_{2}\left(x\right)\\ L_{f}h_{2}\left(x\right) \end{array}\right] \end{array}$$

The system (9) can be represented as

(21) $$\{\begin{array}{l} {\dot{\xi }}_{1}^{1}=\xi_{2}^{1}\text{+}\sum_{i=1}^{6}L_{p_{i}}h_{1}(x)w_{i}\\ {\dot{\xi }}_{2}^{1}=\xi_{3}^{1}\text{+}\sum_{i=1}^{6}L_{p_{i}}L_{f}h_{1}(x)w_{i}\\ {\dot{\xi }}_{3}^{1}=\xi_{4}^{1}\text{+}\sum_{i=1}^{6}L_{p_{i}}L_{f}^{2}h_{1}(x)w_{i}\\ {\dot{\xi }}_{4}^{1}=L_{f}^{4}h_{1}(x)+\sum_{j=1}^{2}L_{g_{j}}L_{f}^{3}h_{1}(x)u_{j}+\sum_{i=1}^{6}L_{p_{i}}L_{f}^{3}h_{1}(x)w_{i}\\ {\dot{\xi }}_{1}^{2}=\xi_{2}^{2}\text{+}\sum_{i=1}^{6}L_{p_{i}}h_{2}(x)w_{i}\\ {\dot{\xi }}_{2}^{2}=L_{f}^{2}h_{2}(x)+\sum_{j=1}^{2}L_{g_{j}}L_{f}h_{2}(x)u_{j}+\sum_{i=1}^{6}L_{p_{i}}L_{f}h_{2}(x)w_{i} \end{array}$$

We define

$$\{\begin{array}{l} e_{1}^{1}=q_{d}-\xi_{1}^{1},e_{2}^{1}={\dot{q}}_{d}-\xi_{2}^{1},e_{3}^{1}={\ddot{q}}_{d}-\xi_{3}^{1}\\ e_{4}^{1}=q_{d}^{\left(3\right)}-\xi_{4}^{1},e_{1}^{2}=\sigma_{d}-\xi_{1}^{2},e_{2}^{2}={\dot{\sigma }}_{d}-\xi_{2}^{2} \end{array}$$

From (21), we can get

(22) $$\{\begin{array}{l} {\dot{e}}_{1}^{1}=e_{2}^{1}-\sum_{i=1}^{6}L_{p_{i}}h_{1}(x)w_{i}\\ {\dot{e}}_{2}^{1}=e_{3}^{1}-\sum_{i=1}^{6}L_{p_{i}}L_{f}h_{1}(x)w_{i}\\ {\dot{e}}_{3}^{1}=e_{4}^{1}-\sum_{i=1}^{6}L_{p_{i}}L_{f}^{2}h_{1}(x)w_{i}\\ {\dot{e}}_{4}^{1}=q_{d}^{\left(4\right)}-L_{f}^{4}h_{1}(x)-\sum_{j=1}^{2}L_{g_{j}}L_{f}^{3}h_{1}(x)u_{j}-\sum_{i=1}^{6}L_{p_{i}}L_{f}^{3}h_{1}(x)w_{i}\\ {\dot{e}}_{1}^{2}=e_{2}^{2}-\sum_{i=1}^{6}L_{p_{i}}h_{2}(x)w_{i}\\ {\dot{e}}_{2}^{2}={\ddot{\sigma }}_{d}-L_{f}^{2}h_{2}(x)-\sum_{j=1}^{2}L_{g_{j}}L_{f}h_{2}(x)u_{j}-\sum_{i=1}^{6}L_{p_{i}}L_{f}h_{2}(x)w_{i} \end{array}$$

and $E\text{=}{\left[\begin{array}{cc} e_{\text{4}}^{\text{1}} & e_{2}^{2} \end{array}\right]}^{T}$,

(23) $$\begin{array}{l} Ė=b\left(x\right)+A\left(x\right)u+D\left(x\right)w \end{array}$$

where

$$\begin{array}{l} D\left(x\right)=\left[\begin{array}{cccccc} -L_{p1}L_{f}^{3}h_{1} & -L_{p2}L_{f}^{3}h_{1} & -L_{p3}L_{f}^{3}h_{1} & -L_{p4}L_{f}^{3}h_{1} & -L_{p5}L_{f}^{3}h_{1} & -L_{p6}L_{f}^{3}h_{1}\\ -L_{p1}L_{f}^{1}h_{2} & -L_{p2}L_{f}^{1}h_{2} & -L_{p3}L_{f}^{1}h_{2} & -L_{p4}L_{f}^{1}h_{2} & -L_{p5}L_{f}^{1}h_{2} & -L_{p6}L_{f}^{1}h_{2} \end{array}\right] \end{array}$$

$$\begin{array}{l} b\left(x\right)=\left[\begin{array}{c} q_{d}^{\left(4\right)}-L_{f}^{4}h_{1}\left(x\right)\\ {\ddot{\sigma }}_{d}-L_{f}^{2}h_{2}\left(x\right) \end{array}\right],\\ A\left(x\right)=\left[\begin{array}{cc} L_{g_{1}}L_{f}^{3}h_{1} & L_{g_{2}}L_{f}^{3}h\\ L_{g_{1}}L_{f}h_{2} & L_{g_{2}}L_{f}h_{2} \end{array}\right]. \end{array}$$

### Composite Control Law Design

Substituting the disturbance *w* in system (22), a NDOB based composite control law is developed as

(24) $$\begin{array}{l} u=A^{-1}\left(x\right)\left[-b\left(x\right)+v+\Gamma \left(x\right)ŵ\right] \end{array}$$

where ŵ is the estimated disturbance by (10), and

$$\begin{array}{l} \Gamma \left(x\right)=\left[\begin{array}{cccccc} \gamma_{11}\left(x\right) & \gamma_{12}\left(x\right) & \gamma_{13}\left(x\right) & \gamma_{14}\left(x\right) & \gamma_{15}\left(x\right) & \gamma_{16}\left(x\right)\\ \gamma_{21}\left(x\right) & \gamma_{22}\left(x\right) & \gamma_{23}\left(x\right) & \gamma_{24}\left(x\right) & \gamma_{25}\left(x\right) & \gamma_{26}\left(x\right) \end{array}\right],\\ \;v=\left[\begin{array}{c} v_{1}\\ v_{2} \end{array}\right] \end{array}$$

$$\begin{array}{l} \gamma_{ij}\left(x\right)=\overset{r_{i}-2}{\underset{k=0}{\sum }}c_{k+1}^{i}L_{p_{j}}L_{f}^{k}h_{i}+L_{p_{j}}L_{f}^{r_{i}-1}h_{i}\left(i=1,2;j=1,2,\cdots \;,6\right) \end{array}$$

$$\begin{array}{l} v_{1}=-c_{0}^{1}e_{\text{1}}^{\text{1}}-c_{1}^{1}e_{\text{2}}^{\text{1}}-c_{2}^{1}e_{\text{3}}^{\text{1}}-c_{3}^{1}e_{\text{4}}^{\text{1}}\\ v_{2}=-c_{0}^{2}e_{1}^{2}-c_{1}^{2}e_{2}^{2} \end{array}$$

where parameters $c_{k}^{i}\left(i=1,2;k=0,1,\cdots \;,r_{i}-1\right)$ are selected such that the polynomials

(25) $$\begin{array}{l} \begin{array}{l} p_{0}^{1}\left(s\right)=c_{0}^{1}+c_{1}^{1}s+\cdots +c_{3}^{1}s^{3}\text{+}s^{\text{4}},\\ p_{0}^{2}\left(s\right)=c_{0}^{2}+c_{1}^{2}s\text{+}s^{2} \end{array} \end{array}$$

are Hurwitz stable.

**Figure 2:**
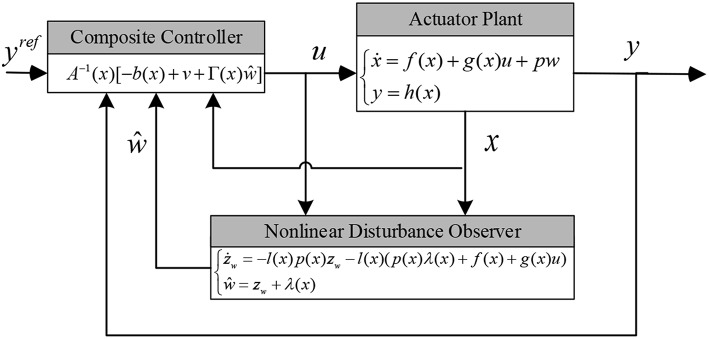
The schematic diagram for the NDOB based composite controller.

The schematic diagram of the proposed NDOB-based control design can be expressed in Figure 2. In order to prove that the control law is effective on disturbance, the disturbance estimation should be replaced by real disturbance.

(26) $$\begin{array}{l} u=A^{-1}\left(x\right)\left[-b\left(x\right)+v+\Gamma \left(x\right)w\right] \end{array}$$

Substituting (26) into (23), we can get

(27) $$\begin{array}{l} {ė}_{r_{i}}^{i}=v_{i}+\overset{n}{\underset{k=1}{\sum }}\left(\gamma_{ik}-L_{p_{k}}L_{f}^{r_{i}-1}h_{i}\right)w_{k} \end{array}$$

Combining (27) with (22), the error dynamic equation can be rewritten as

(28) $$\{\begin{array}{l} {\dot{e}}^{i}=A^{i}e^{i}+D^{i}(x)w\\ \;e_{1}^{i}=C^{i}e^{i} \end{array}$$

where

$$\begin{array}{l} A^{i}=[\begin{array}{ccccc} 0 & 1 & 0 & \cdots & 0\\ 0 & 0 & 1 & \cdots & 0\\ \vdots & \vdots & \vdots & \ddots & \vdots \\ 0 & 0 & 0 & \cdots & 1\\ -c_{0}^{i} & -c_{1}^{i} & -c_{2}^{i} & \cdots & -c_{r_{i}-1}^{i} \end{array}], \end{array}$$

$$\begin{array}{l} D^{i}\left(x\right)=[d_{1}^{i},\ldots ,d_{\text{6}}^{i}], \end{array}$$

$$\begin{array}{l} d_{j}^{i}=\left[\begin{array}{c} -L_{pj}h_{i}\\ -L_{pj}L_{f}h_{i}\\ \vdots \\ \overset{r_{i}-2}{\underset{k=0}{\sum }}c_{k+1}^{i}L_{pj}L_{f}^{k}h_{i} \end{array}\right]1\leq j\leq 6 \end{array}$$

$$\begin{array}{l} B^{i}={\left[0,0,\cdots \;,1\right]}_{1\times r_{i}}^{T}\\ C^{i}={\left[1,0,\cdots \;,0\right]}_{1\times r_{i}} \end{array}$$

Equation (28) can be written as

(29) $$\begin{array}{l} \begin{array}{l} e_{\text{1}}^{i}=C^{i}{\left(A^{i}\right)}^{-1}\left[{\dot{\xi }}^{i}-D^{i}\left(x\right)w\right]\\ \;=C^{i}{\left(A^{i}\right)}^{-1}{\dot{\xi }}^{i}-C^{i}{\left(A^{i}\right)}^{-1}D^{i}\left(x\right)w \end{array} \end{array}$$

in which

(30) $$\begin{array}{l} C^{i}{\left(A^{i}\right)}^{-1}D^{i}\left(x\right)\text{=0} \end{array}$$

and

(31) $$\begin{array}{l} e_{\text{1}}^{i}=C^{i}{\left(A^{i}\right)}^{-1}{\dot{\xi }}^{i} \end{array}$$

We can see that the disturbance has been compensated according to (31) and $\lim_{t\to \infty }e_{1}^{i}\left(t\right)=0$.

### System Stability Analysis

**Theorem 1**. If the following conditions are satisfied, the system (9) is locally **ISS** around *x*_0_:

- The parameters *c*^*i*^ in the NDOBC law (24) are chosen such that the polynomials (25) are Hurwitz stable;
- The disturbance gain is chosen to keep the function *g*(*x*)*A*^−1^(*x*)Γ(*x*) + *p*(*x*) continuously differentiable at *x*_0_;
- The observer gain is chosen such that the system (13) is asymptotically stable.

**Proof:** Substituting the NDOBC law (24) into the dynamic system (9), we can get the closed-loop system:

(32) $$\{\begin{array}{l} \dot{x}=G(x,e_{w},w)\\ {\dot{e}}_{w}=H(e_{w},\dot{w}) \end{array}$$

where

$$\begin{array}{l} \begin{array}{l} G\left(x,e_{w},w\right)=f\left(x\right)+\left[g\left(x\right)A^{-1}\left(x\right)\Gamma \left(x\right)+p\left(x\right)\right]w\\ +g\left(x\right)A^{-1}\left(x\right)\left[-b\left(x\right)+v-\Gamma \left(x\right)e_{w}\right] \end{array} \end{array}$$

and

(33) $$\begin{array}{l} H\left(e_{w},ẇ\right)=-l\left(x\right)p\left(x\right)e_{w}+ẇ \end{array}$$

Based on the new coordinate transformation $\left[e_{\text{1}}^{\text{1}},e_{\text{2}}^{\text{1}},e_{\text{3}}^{\text{1}},e_{\text{4}}^{\text{1}},e_{1}^{2},e_{2}^{2}\right]$, the closed-loop system (26) includes the system *ẋ* = *f*(*x*) + *g*(*x*)*u* and the control law *u* = *A*^−1^(*x*)(−*b*(*x*) + *v*) is represented by

(34) $$\begin{array}{l} ė=Ae \end{array}$$

where

$$\begin{array}{l} A=\left[\begin{array}{cccccc} 0 & 1 & 0 & 0 & 0 & 0\\ 0 & 0 & 1 & 0 & 0 & 0\\ 0 & 0 & 0 & 1 & 0 & 0\\ -c_{0}^{1} & -c_{1}^{1} & -c_{2}^{1} & -c_{3}^{1} & 0 & 0\\ 0 & 0 & 0 & 0 & 0 & 1\\ 0 & 0 & 0 & 0 & -c_{0}^{2} & -c_{1}^{2} \end{array}\right]. \end{array}$$

It can be concluded that the system (34) is asymptotically stable at equilibrium *x* = 0.

Let $X={\left[x^{T},{e_{w}}^{T}\right]}^{T}$, the system (34) is given by

(35) $$\begin{array}{l} Ẋ=\underset{-}{G}\left(X\right)+\underset{-}{H}\left(X\right)\underset{-}{w} \end{array}$$

where

$$\begin{array}{l} \underset{-}{G}\left(x\right)=\left[\begin{array}{c} G\left(x,e_{w},0\right)\\ H\left(e_{w},0\right) \end{array}\right],\\ \underset{-}{H}\left(X\right)=\left[\begin{array}{cc} g\left(x\right)A^{-1}\left(x\right)\Gamma \left(x\right)+p\left(x\right) & 0\\ 0 & I_{n\times n} \end{array}\right],\;\underset{-}{w}=\left[\begin{array}{c} w\\ ẇ \end{array}\right]. \end{array}$$

Based on the theorem of the asymptotic stability (Khalil, 2002), the system $Ẋ=\underset{-}{G}\left(x\right)$ is locally asymptotically stable at *X* = 0, according to the condition (ii), the system (34) is locally ISS.

## Simulation Results

**Figure 3:**
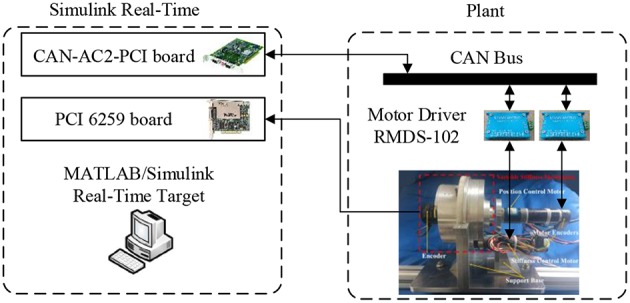
Control hardware of the SVSA.

To demonstrate the proposed NDOBC approach and point out its performance properties, a comparative simulation study with the control law has been conducted for the SVSA under external load disturbances as presented in Figure 3. The SVSA is first considered to verify and clarify the operation of the developed controller. The specifications of the SVSA given in Table 1 is used for simulation. We set the parameters for nominal model *M*_*n*_ = 0.0153*kg* ▪ *m*^2^, *B*_1*n*_ = 0.0284*kg* ▪ *m*^2^, *B*_2*n*_ = 0.019*kg* ▪ *m*^2^, *D*_*n*_ = 0.007*N* ▪ *m* ▪ *s*, *D*_1*n*_ = 0.007*N* ▪ *m* ▪ *s*, *D*_2*n*_ = 0.003*N* ▪ *m* ▪ *s*, and the initial variables are set as *x*(0) = [0 0 0 0 0 0]. To make a comparison, a feedback linearization-based (FL) controller is selected as a baseline controller, which is given by

(36) $$\begin{array}{l} u=A^{-1}\left(x\right)\left(v-b\left(x\right)\right) \end{array}$$

The unknown external disturbances are given by

$$\{\begin{array}{l} w_{1}(t)\text{=}w_{3}(t)\text{=}w_{5}(t)=\text{0}\\ w_{2}(t)=w_{4}(t)=w_{6}(t)=\{\begin{array}{c} 0,t<\text{5}\\ 2,\;t\geq \text{5}. \end{array} \end{array}$$

The results of the comparison between the baseline controller and the NDOB controller are illustrated.

### Tracking Under Fixed Stiffness

Sinusoidal trajectory of the actuator position with frequency of 0.2 Hz and amplitude of 60° was taken. A 3 kg load disturbance is introduced at 5 s. The purpose of the simulation is to test the performance of the controller to track the trajectory at two different stiffness conditions, which is low stiffness (15 Nm/rad) and high stiffness (60 Nm/rad), respectively.

**Figure 4:**
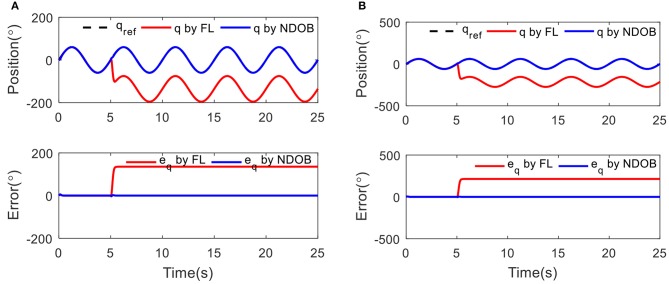
The position tracking results under 3 kg load disturbance at 5 s with different condition: **(A)** low stiffness (15 Nm/rad) and **(B)** high stiffness (60 Nm/rad).

Figure 4 shows that the proposed NDOBC approach exhibits promising disturbance attenuation and reference tracking performance. It is also observed that the tracking trajectory under the NDOBC is overlapped with the baseline control method during the first 5 s when there is no disturbance acted on the system, but poor tracking performance after loading, which proves that the property of the NDOBC method. In addition, the stiffness has little effect on the tracking performance under constant stiffness condition.

### Tracking With Variable Stiffness

**Figure 5:**
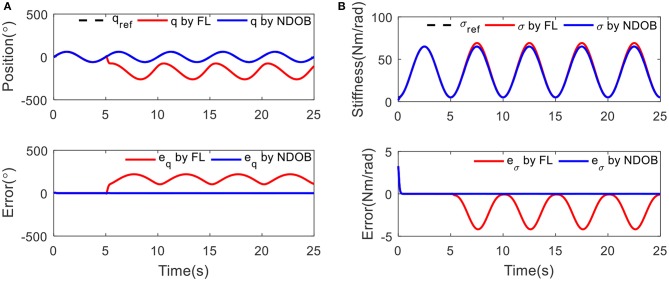
The tracking results under 3 kg load disturbance at 5 s: **(A)** position tracking and **(B)** stiffness tracking.

Sinusoidal trajectory was taken under variable stiffness condition, where σ(*t*) = 35 + sin(2π*ft* + 1.5π)with the frequency of 0.2 Hz. In Figure 5, it can be seen that both the position and stiffness tracking errors are small without external load for two controllers. After loading 3 kg at 5 s, position and stiffness tracking errors increase with FL control, but the NDOBC performance is better than the baseline. The tracking error is small, which means the disturbance can be compensated.

## Experimental Results

To further verify the robustness of the controller, an experimental procedure was carried out on the SVSA platform. Two DC motors (RE50, 60W and RE25, 20W, Maxon motor) were selected as the driving modules. Two motor drivers (RMDS-102, ShenZhen RoboModule Technology Co., China) were used to control the motors. Encoders with 500 pluses per revolution were installed to measure the motor position. An Omron encoder (E6B2-CWZ1X) was utilized to measure the deflection angle of the SVSA. A Simulink real-time control system was built based on MATLAB/RTW in xPC target environment using CAN-AC2-PCI board (as shown in Figure 3). The angles of the encoders were collected via a data acquisition card (PCI-6259, National Instruments Corp., TX) to MATLAB/RTW control system. The communication between the real-time system and the Plant is through CAN Bus.

### Tracking With Fixed Stiffness

**Figure 6:**
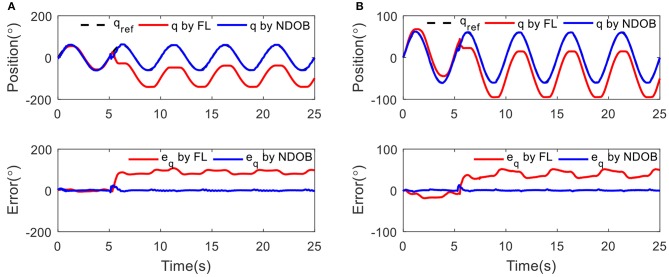
The position tracking results under 3 kg load disturbance at 5 s with different condition: **(A)** low stiffness (15 Nm/rad) and **(B)** high stiffness (60 Nm/rad).

Sinusoidal tracking experiments with frequency of 0.2 Hz and amplitude of 60° at two different conditions, low stiffness (15 Nm/rad) and high stiffness (60 Nm/rad), were conducted. Figure 6 shows the position tracking and output errors for both controllers in the presence of external load disturbance at 5 s. The robustness of the NDOB controller is obvious because the error continues to reduce despite the external load. The disturbance is also clearly shown in the output error. It shows that the NDOB control can achieve better position tracking results within the first 5 s. The baseline control performance is deteriorated when adding the 3 kg load. In addition, compared with the low stiffness condition, we can find that the tracking error is reduced in high stiffness, which means external disturbances have less impact on the position tracking error at high stiffness. This can be explained that the deflection angle in low stiffness is larger than that of the high stiffness condition. However, compared with the simulation results, the experimental data exhibit small oscillations during the tracking.

### Tracking With Variable Stiffness

**Figure 7:**
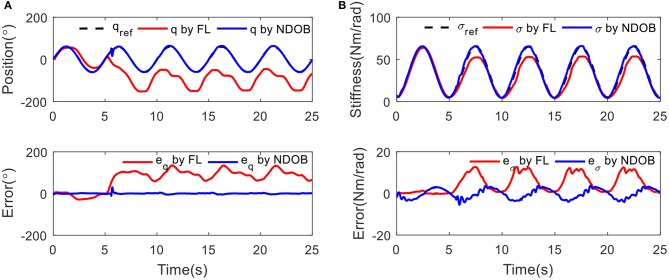
The tracking results under 3 kg load disturbance at 5 s: **(A)** position tracking and **(B)** stiffness tracking.

Secondly, the controller performance has been tested while tracking a sine wave reference on continuous position and stiffness. Three kilograms load is applied at 5 s. The stiffness σ(*t*) = 35 + sin(2π*ft* + 1.5π) has been adjusted with the frequency of 0.2 Hz. Figure 7 shows the position and stiffness tracking results with and without external load disturbance for two controllers. The NDOB control achieved better results than the FL controller. The position tracking error suddenly increases due to the external disturbances at 5 s. In stiffness tracking, there is no obvious change under the disturbance compensation algorithm while the error increases for FL control.

## Conclusion

This paper proposed a NDOBC to attenuate the model uncertainties and external disturbances for a class of SVSA. Simulation and experimental results verify the ability of the proposed approach to cope with load disturbance by showing remarkable control performances for both position and stiffness tracking. The stability of the composite controller has been proved by the tracking results. Future work will focus on other non-linear composite adaptive control designs for the SVSA to solve the input saturation and unmodeled dynamics (Pan and Yu, 2016; Sun N. et al., 2018) and the application of this actuator to the design of variable stiffness robots in real-world applications.

## Data Availability

The raw data supporting the conclusions of this manuscript will be made available by the authors, without undue reservation, to any qualified researcher.

## Author Contributions

ZG: theoretical analysis and writing paper. JS: VSA design. JL: guide doing experiment. YP: guide control plan. XX: guide writing paper.

### Conflict of Interest Statement

The authors declare that the research was conducted in the absence of any commercial or financial relationships that could be construed as a potential conflict of interest.
